# Strategies for Remodeling the Tumor Microenvironment Using Active Ingredients of Ginseng—A Promising Approach for Cancer Therapy

**DOI:** 10.3389/fphar.2021.797634

**Published:** 2021-12-22

**Authors:** Mo Li, Xin Wang, Ying Wang, Shunchao Bao, Qing Chang, Linlin Liu, Shuai Zhang, Liwei Sun

**Affiliations:** ^1^ Department of Radiotherapy, The Second Hospital of Jilin University, Changchun, China; ^2^ Department of Thyroid Surgery, The Second Hospital of Jilin University, Changchun, China; ^3^ Department of Neurology, China-Japan Union Hospital of Jilin University, Changchun, China; ^4^ Department of Breast Surgery, The Second Hospital of Jilin University, Changchun, China; ^5^ Jilin Ginseng Academy, Changchun University of Chinese Medicine, Changchun, China; ^6^ Research Center of Traditional Chinese Medicine, The Affiliated Hospital to Changchun University of Chinese Medicine, Changchun, China

**Keywords:** tumor microenvironment, tumor angiogenesis, tumor stem cell, immune response, *Panax ginseng* (C.A. Meyer), cancer therapy

## Abstract

The tumor microenvironment (TME) plays a key role in promoting the initiation and progression of tumors, leading to chemoradiotherapy resistance and immunotherapy failure. Targeting of the TME is a novel anti-tumor therapeutic approach and is currently a focus of anti-tumor research. *Panax ginseng* C. A. Meyer (ginseng), an ingredient of well-known traditional Asia medicines, exerts beneficial anti-tumor effects and can regulate the TME. Here, we present a systematic review that describes the current status of research efforts to elucidate the functions and mechanisms of ginseng active components (including ginsenosides and ginseng polysaccharides) for achieving TME regulation. Ginsenosides have variety effects on TME, such as Rg3, Rd and Rk3 can inhibit tumor angiogenesis; Rg3, Rh2 and M4 can regulate the function of immune cells; Rg3, Rd and Rg5 can restrain the stemness of cancer stem cells. Ginseng polysaccharides (such as red ginseng acidic polysaccharides and polysaccharides extracted from ginseng berry and ginseng leaves) can regulate TME mainly by stimulating immune cells. In addition, we propose a potential mechanistic link between ginseng-associated restoration of gut microbiota and the tumor immune microenvironment. Finally, we describe recent advances for improving ginseng efficacy, including the development of a nano-drug delivery system. Taken together, this review provides novel perspectives on potential applications for ginseng active ingredients as anti-cancer adjuvants that achieve anti-cancer effects by reshaping the tumor microenvironment.

## Introduction

Efforts to develop anti-cancer therapies no longer focus specifically on targeting tumor cells themselves, since cancer progression is regulated by the interaction between tumor cells and the tumor-site environment. The tumor microenvironment (TME) is a complex tumor ecosystem that are dominated by tumor and consists of tumor cells, stromal cells, immune cells, and the extracellular matrix (Wang et al., 2021; Zhang et al., 2021). The overall complexity of an anti-tumor treatment depends on interactions between the TME and tumor that support tumor growth and heterogeneity, as well as tumor invasion, metastasis, immune escape, and resistance to radiotherapy and chemotherapy (Cheng et al., 2020; Abou Khouzam et al., 2021). In view of the important role of the TME in tumor development, tumor treatment approaches have evolved from traditional measures aimed at eliminating tumor cells to multi-pronged comprehensive treatment measures focused on eliminating both tumor cells and the TME. Therapeutic strategies targeting the TME include enhancement of anti-tumor immunity, inhibition of tumor angiogenesis, administration of anti-inflammatory agents, and blockage of communication between tumor cells and the extracellular matrix (Pitt et al., 2016). A variety of drugs that target the TME are currently in widespread clinical use. For example, antibody treatments are used to enhance the anti-tumor immune response by blocking negative immunomodulatory effects on “immune checkpoints” that are induced by tumor or immunosuppressive cells within the TME. Such antibodies include anti-CTLA-4 antibodies that restore costimulatory signaling of cytotoxic T lymphocytes (CTLs) and anti-PD-1/PD-L1 antibodies that block PD-1/PD-L1 inhibition of activated CTL function. Meanwhile, drugs targeting tumor angiogenesis are also available that include antibodies targeting the VEGF/VEGFR axis, which block tumor-site endothelial cell angiogenesis and promote vascular normalization. Therefore, targeting of the TME is a novel and promising strategy for the development of anti-tumor drugs.

*Panax ginseng* C.A. Meyer, known as “the king of herbs,” has been used in Asian medicine for thousands of years to treat illness and was also used primarily as an energy and body balance tonic in ancient times. Nowadays, ginseng has been shown to have benefits for relieving a variety of disorders, such as inflammation, infection, fatigue, effects of aging, and cancer. Active ingredients of ginseng, such as ginsenosides and polysaccharides, have been shown to possess significant anti-tumor activities (Sun et al., 2017; Ahuja et al., 2018; Li X. et al., 2020; Guo et al., 2021). Ginsenosides induce tumor cell death by inducing initiation of programmed death pathways or inhibiting tumor proliferation by interfering with tumor cell cycle and metabolic pathways. In addition, ginsenosides effectively inhibit tumor cell invasion and metastasis, while ginseng polysaccharides have been shown to induce tumor cell apoptosis and inhibit tumor cell metastasis. Furthermore, ginseng may counter immunosuppressive TME effects by modulating immune cell differentiation and functions and regulating immune checkpoints to restore anti-tumor immune functions. Collectively, these results indicate that ginseng anti-tumor efficacy may depend on abilities of its constituents to regulate multiple targets within the TME. In this review, molecular mechanisms underlying TME regulation by ginseng active ingredients are reviewed.

## The Role of Ginsenosides in Inhibiting Tumor Angiogenesis

Under normal physiological conditions, blood vessel formation is a tightly controlled process. However, during the process of tumor proliferation, the tumor-dominated microenvironment promotes aberrant angiogenesis by breaking the vascular homeostatic balance (Weis and Cheresh, 2011). A large number of pro-angiogenic and anti-angiogenic factors participate in vascular homeostasis. When these factors are in equilibrium, the vascular system remains stable and endothelial cells do not proliferate. By contrast, when the “angiogenesis switch” is turned on, the vascular homeostatic balance becomes disrupted and tips in favor of pro-angiogenesis (Hanahan and Folkman, 1996; Bergers and Benjamin, 2003; Lugano et al., 2020). Bone marrow-derived endothelial progenitor cells (EPCs) recruited by tumors participate in the pathological neovascularization and growth of early tumors by modulating the angiogenic switch (Nolan et al., 2007; Oh et al., 2007; Plummer et al., 2013; Bonfim-Silva et al., 2017; Xu et al., 2017). Moreover, hypoxic conditions, a hallmark of the TME, are closely related to initiation of tumor angiogenesis, whereby the ubiquitin-proteasome pathway of hypoxia inducible factor-1 (HIF-1) is inhibited under hypoxic conditions, which leads to intracellular HIF-1 complex accumulation that promotes initiation of transcription of pro-angiogenic genes. Pro-angiogenic factors and their homologous receptors effectively co-operate to promote angiogenesis within tumors during which vascular endothelial growth factor and its receptor (VEGF/VEGFR) system play a pivotal role (Weis and Cheresh, 2005; Shibuya, 2013; Ribatti and Tamma, 2019). Furthermore, the leakage and collapse of tumor blood vessels can exacerbate hypoperfusion and hypoxia, leading to increased secretion of VEGF. Meanwhile, other angiogenesis-related factors and molecules (e.g., ANG1, FGF2, PDGF, ephrins, MMPs, etc.) may also contribute to the formation of a defective vascular network in tumors (Lugano et al., 2020) that may explain why neo-vessels in the TME possess abnormal morphology and network structure and have increased blood vessel permeability. In turn, aberrant angiogenesis supports tumor growth, invasion, and metastasis and may intensify the hypoxic microenvironment surrounding the tumor to promote tumor proliferation.

Anti-angiogenesis therapies, which mainly target the VEGF signaling pathway, have been approved for treatment of a variety of tumors (Lugano et al., 2020; Shibuya, 2013), although drug development is ongoing due to drug resistance and adverse reactions. Natural herbs such as ginseng are increasingly being viewed as potential anti-angiogenesis drugs, including various ginsenoside monomers that have been found to inhibit angiogenesis in tumors (Table 1). For example, ginsenoside Rg3 was found to inhibit EPCs differentiation, proliferation, and migration by suppressing VEGF-dependent p38/ERK and Akt/eNOS signal pathways *in vitro* (Kim et al., 2012a; Kim et al., 2012b), while also attenuating neo-vessel formation and mobilization of EPCs *in vivo*, leading to delayed tumor progression and angiogenesis (Kim et al., 2012a). According to Tang *et al.*, ginsenoside Rg3 appeared to decrease microvessel density levels in colorectal cancer xenografts by downregulating expression of certain angiogenesis-related genes, such as *CSF3*, *FGF2*, *MMP1*, and *PGF* (Tang et al., 2018). Under TME hypoxic conditions, HIF-1 complex, of which HIF-1α is a key component, could further activate various downstream angiogenic factors in tumor cells, such as VEGF. In other studies, ginsenoside Rg3 has been shown to inhibit angiogenesis in a variety of tumor models and was shown to inhibit hypoxia-induced VEGF expression in tumor cells (Ge et al., 2014; Li and Qu, 2019; Meng et al., 2019) through regulation of various pathways, such as Akt, ERK, JNK, and STAT3 signaling pathways (Chen et al., 2010; Meng et al., 2019). Another ginsenoside, Rd, was reported to exert anti-angiogenic effects by mitigating VEGF-induced migration, invasion, and capillary formation by human umbilical vascular endothelial cells (HUVECs) and by reducing CD31-positive capillary formation in tumors by inhibiting both VEGF/VEGFR2 signaling cascade pathways Akt/mTOR/p70S6K and HIF-1α expression (Zhang et al., 2017). Meanwhile, ginsenosides Rb1 and Ro have been shown to inhibit HUVEC cell formation into tube-like structures (Lu et al., 2017; Zheng et al., 2019), while the end metabolite of 20(S)-protopanaxadiol-type ginsenosides metabolism, PPD, exerted a pro-apoptotic effect on HUVECs (Wang X. et al., 2019) and ginsenoside Rh2 downregulated tumor cell expression of VEGF and MMPs (Li H. et al., 2018; Zhang et al., 2020).

**TABLE 1:** Effects of Ginsenosides on angiogenesis.

Components	Cell/animal model	Effects	Main mechanisms	Ref.
Ginsenoside Rg3	*In vitro*, EPCs; *In vivo*, Lewis lung carcinoma (LLC) tumor-bearing murine model	Could suppress EPCs proliferation, migratory ability and tubular formation ability *in vitro*, and decrease number of peripheral EPCs and tumor capillary *in vivo*	Suppressing VEGF dependent p38 and ERK signal pathways	Kim et al. (2012a)
Ginsenoside Rg3	*In vitro*, cord blood-derived CD34 stem/progenitor cells	Could attenuating EPC differentiation of human cord blood derived CD34-positive stem cells	Inhibiting VEGF dependent Akt/eNOS pathways	Kim et al. (2012b)
Ginsenoside Rg3	*In vitro*, colorectal cancer cells(CRC) LoVo; *in vivo*, LoVo orthotopic xenograft murine model	Could decrease microvessel density (MVD) levels	Downregulating expression of several pro-angiogenic genes	Tang et al. (2018)
Ginsenoside Rg3	*In vitro*, gastric cancer cell line BGC823	Could inhibit expression of VEGF	Downregulating expression of HIF-α	Li and Qu, (2019)
Ginsenoside Rg3	*In vitro* and *in vivo*, a highly metastatic subline of murine B16 melanoma cells	Could decrease the number of vessels oriented toward the tumor lesions, and reduce vascular endothelial cell proliferation and migration	Downregulating expression of VEGF by inhibiting Akt and ERK pathways	Meng et al. (2019)
Ginsenoside Rd	*In vitro*, HUVECs; *in vivo*, breast cancer cell line MDA-MB-231 mouse model	Could inhibit VEGF-dependent migration, vascularization and viability and angiogenesis activity of HUVECs, and prevent tumor angiogenesis	Inhibiting expression of HIF-α and VEGF/VEGFR through Akt/mTOR/p70S6K signaling pathway	Zhang et al. (2017)
Ginsenoside Rk3	*In vivo*, human NSCLC H460 xenograft mouse model, chick embryo chorioallantoic membrane (CAM) model	Could decrease the expression of endothelial cell marker CD34 in tumor tissues and inhibit angiogenetic activity in CAM model	Not clear	Duan et al. (2017)
20(S)-protopanaxadiol	*In vitro*, HUVECs	Could induce endoplasmic reticulum stress and apoptosis of HUVECs	Inducing PERK-eIF2-ATF4-CHOP signaling pathway.	Wang X. et al. (2019)
Ginsenoside Rb1	*In vitro*, HUVECs	Could suppressing the formation of endothelial tube-like structures	Increasing expression of PEDF via activating PPAR-γ/miR-33a pathway	Lu et al. (2017)
Ginsenoside Ro and its metabolites	*In vitro*, HUVECs	Could inhibit tube formation of HUVECs.	Not clear	Zheng et al. (2019)
Ginsenoside F1 and Rh1	*In vitro* and *in vivo*, HUVECs and human retinal microvascular endothelial cells(HRMECs)	Could inhibit VEGF-induced vascular leakage	Targeted suppressing NR4A1 expression and transcriptional activity	Kang et al. (2019)

**FIGURE 1:**
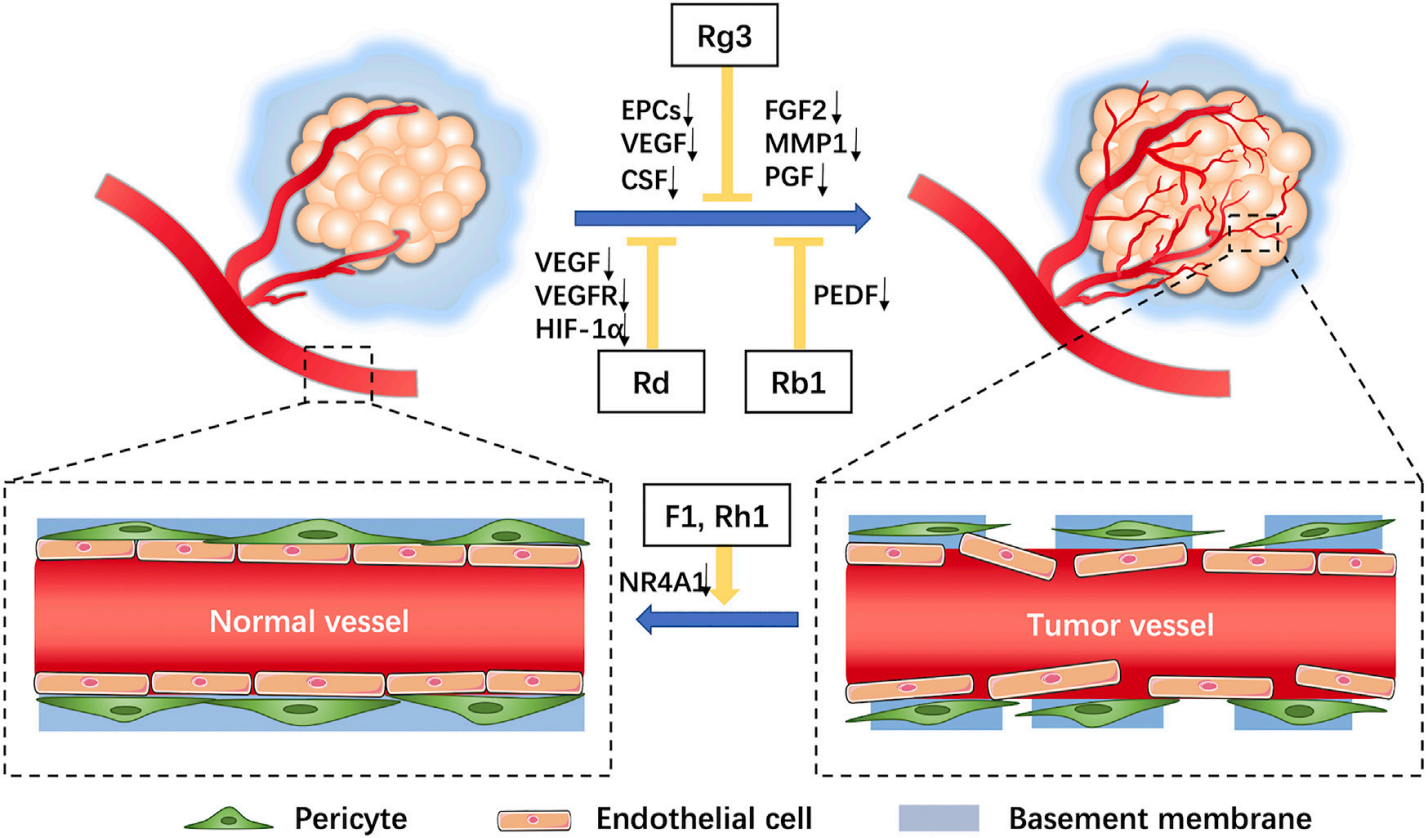
Characteristics of tumor vessels and roles of ginsenosides on tumor angiogenesis. In the tumor microenvironment, tumor blood vessels comprise a tortuous, over-branched, and disordered vascular network structure with increased vascular permeability (increased endothelial cell gaps, loss of pericyte coverage, and incomplete basement membrane). Ginsenosides can inhibit effects of angiogenic factors on tumor angiogenesis and facilitate vascular normalization.

The tumor vascular network is characterized by dilated, twisted, and disordered immature vessels lacking parietal cells (Figure 1) that exhibit hyperpermeability, poor perfusion, and increased hypoxia (Viallard and Larrivée, 2017). Recent data have demonstrated that some ginsenosides, such as F1 and Rh1, act to reduce vascular leakage induced by VEGF by suppressing mRNA transcription and protein expression of NR4A1 (Kang et al., 2019), leading to vascular normalization that prevents tumor cell extravasation and metastasis.

## Ginseng May Regulate the Tumor Immunosuppressive Microenvironment

An important component of the TME, infiltrating immune cells, can be classified as tumor-suppressors and tumor-promotors based on functions of these tumor-associated cells (Lei et al., 2020). In general, although immune cells within the body are capable of recognizing and killing tumor cells, anti-tumor immune responses in the TME are suppressed due to direct or indirect tumor interference with, and/or inhibition of, functions of tumor-antagonizing immune cells acting via many mechanisms. Ultimately, dysfunctional immunoregulation within the TME promotes proliferation and differentiation of tumor-promoting immune cells that eventually lead to abnormal immunosurveillance and tumor cell immune escape (Li W. et al., 2020; Lei et al., 2020; Liskova et al., 2020). Several active components of ginseng may exert anti-tumor effects by interfering with the differentiation and maturation of tumor-promoting immune cells, leading to reversal of the inhibitory phenotype of tumor-antagonizing immune cells and restoration of anti-tumor functions of innate (Table 2) and acquired immune cells.

**TABLE 2:** Effects of ginseng on innate immune cells.

Components	Cell/animal model	Effects	Main mechanisms	Ref.
**Macrophages**				
Rg3-LPs	*In vitro* and *in vivo*, C6 murine glioma cells and tumor-bearing murine models	Could enhance PTX cytotoxicity and apoptosis in C6 cells, prolong median survival time of tumor-bearing mice/rats	A synergistic effect on TAM repolarization with PTX; decreasing the numbers of granulocyte-like myeloid derived suppressor cells in tumor microenvironment	Zhu et al. (2021)
Ginsenoside Rh2	*In vitro*, the murine macrophage-like cell line RAW264.7 and NSCLC cells; *in vivo*, LLC-bearing murine model	Could inhibit the growth and migration of human lung cancer cells	Inducing repolarization of TAM to M1-like phenotype; downregulating M2 macrophages-induced secretion and expression of VEGF-C, MMP2, and MMP9 in NSCLC cells; decreasing the VEGF-C and marker of M2-like phenotype expression	Li H. et al. (2018)
Ginsenoside Rp1	*In vitro*, the J774A.1 macrophage cells and CT26 colon cancer cells	Could decrease the migration activities of colon cancer cells and prolong the survival rates of tumor-bearing mice	Inhibiting ionizing radiation enhanced LPS-stimulated NO synthesis and IL-1β production in macrophages; radioprotective effects on J774A.1 via inhibiting MAPK and Akt pathways	Baik et al. (2020)
Red ginseng acidic polysaccharide	*In vitro* and *in vivo*, murine melanoma B16 cells and tumor-bearing model of C57BL/6 mice	Could enhance the tumoricidal activity of murine peritoneal macrophages against B16 cells	Increasing production of IL-1, IL-6, TNF-α and NO via activating NF-κB pathway when combine with IFN-γ treatment	Choi et al. (2008)
Ginseng neutral polysaccharide fraction	Sarcoma-180 cells and tumor-bearing ICR mice	Could inhibit tumor growth	Augmenting macrophage phagocytosis and stimulating production of TNF-α and NO	Ni et al. (2010)
Heat-processed ginseng	*In vitro*, the murine macrophage-like cell line RAW264.7	Could enhance the macrophage activation	Increasing cytokine production and MHC expression in macrophage cells and activating MAPKs and NF-κB pathways	Shin et al. (2018)
**DCs**				
Ginsenoside Rg3	*In vitro*, LLC and melanoma cell lines B16F10	Could suppress growth of tumor cells and increase DC uptake function on tumor cells	Increasing immunogenic cell death of tumor cells; suppressing secretion of IFN-γ and inducing secretion of IL-6, TNF-α and TGF-β1 of tumor cells.	Son et al. (2016)
M1 and M4	*In vitro*, human peripheral blood mononuclear cells (PBMCs) derived DCs	Could promote DCs maturation, enhance stimulatory efficiency on naïve T cells differentiating towards Th1 type and augment the cytotoxicity of CD8+T cells	Increasing immune DCs surface expression of CD80, CD83, CD86 and HLA-DR; modulating murine DCs to secrete more IFN-γ and less IL-4 cytokines	Takei et al. (2004)
Neutral ginseng polysaccharides(NGP)	*In vitro*, bone marrow dendritic cells (BMDCs) of C57BL/6 mice	Could promote DCs maturation and increase proliferation of T cells	Increasing expression of CD40, CD80, CD83, CD86 and MHC-II on BMDCs and cytokines IL-12p70 and TNF-α secretion	Meng et al. (2013)
Ginseng polysaccharides	*In vivo*, NSCLC patients	Could improve quality of life when treatment with DCs	Increasing the level of Th1 cytokines (INF-γ, IL-2) and the ratio of Th1/Th2 cytokines (INF-γ/IL-4, IL-2/IL-5); decreasing the level of Th2 cytokines.	Ma et al. (2014)
Acidic ginseng polysaccharides (from red ginseng)	*In vitro*, BMDCs of C57BL/6 mice	Could induce DCs maturation	Increasing surface markers, MHC II, CD80, CD86, CD83 and CD40 on the DCs, and inducing secretion of higher level of IL-12 and low level of TNF-α	Wang et al. (2013)
Ginseng berry extract	*In vitro*, BMDCs of C57BL/6 mice; *in vivo*, B16F10-bearing murine model	Could stimulate BMDCs maturation, increase spleen DCs proportion and activation; could enhance anti-cancer immune response as an immune adjuvant	Upregulating co-stimulatory molecules and production of pro-inflammatory cytokines of BMDCs and spleen DCs via TLR4 and MyD88 signaling pathways	Zhang et al. (2015)
**NK cells**				
Ginsenoside F1	*In vitro*, human NK cells isolated from PBMCs; *in vivo*, lymphoma and melanoma implantation murine model	Could promote the cytotoxicity activity	Increasing the levels of NK cells cytotoxic effector molecules(perforin and granzyme B) and activating signaling downstream (PI3K/Akt) of NK cell-activating receptors (NKG2D and 2B4) and IGF-1 pathway	Kwon et al. (2018)
M4	*In vitro* and *in vivo*, murine melanoma cells B16-BL6 and murine tumor-bearing model of C57BL/6 mice	Could Inhibit tumor growth and metastasis	Stimulating splenic NK cells cytotoxic activity against tumor cells	Hasegawa et al. (2002)
Ginseng berry polysaccharide portion (GBPP)	*In vitro*, splenic NK cells of tumor-bearing BALB/c mice; *in vivo*, B16-BL6 melanoma lung cancer metastasis model of BALB/c mice	Could reduce tumor metastasis colonies in lung	Promoting NK cell cytotoxicity via the release of IFN-γ and granzyme B; enhancing macrophages and cytotoxic T lymphocytes activity	Lee et al. (2019a), Lee et al. (2019b)
Ginseng leaves polysaccharide fraction	*In vitro*, splenic NK cells of tumor-bearing BALB/c mice; *In vivo*, colon 26-M3.1 carcinoma cells lung cancer metastasis model of BALB/c mice	Could inhibit lung metastasis	Activating macrophages and NK cells	Shin et al. (2017)
Ginseng fruits polysaccharide	*In vitro*, splenic NK cells of tumor-bearing C57BL/6 mice and LLC cells; *in vivo*, LLC-bearing model	Could Inhibit tumor growth and metastasis	Enhancing the NK cell-mediated cytotoxicity	Wang et al. (2015)

### Ginseng Enhancement of the Innate Immune Response

#### Macrophages

Macrophages exhibit developmental plasticity and can differentiate into pro-inflammatory (M1) and anti-inflammatory (M2) phenotypes according to different pathological environments (Wang Y. et al., 2019). Available studies indicate that tumor-associated macrophages (TAMs) derived from circulating monocytes and myeloid-derived suppressor cells (MDSCs) mainly possess characteristics and phenotypes of pro-tumorigenic M2-polarized macrophages that promote tumor angiogenesis, enhance tumor metastasis, and inhibit anti-tumor T cell immunity (Kim and Bae, 2016). Thus, strategies that Increase the M1/M2 ratio or inhibit effects of M2-polarized cells are promising anti-tumor therapeutic approaches that work by targeting the TME.

Recent studies have shown that ginsenosides may help to regulate the two subpopulations of TAMs. One such study declared that ginsenoside Rg3 treatment led to an improved anti-tumor effect based on the re-education and conversion of TAMs from an M2 phenotype to an M1 phenotype when Rg3 was delivered to cells within liposomes that were also loaded with paclitaxel (Rg3-PTX-LPs) (Zhu et al., 2021). Another ginsenoside, Rh2, has also been shown to alter the TME by inducing conversion of TAMs from an M2 to an M1 phenotype that, in turn, prevented tumor cell migration and secretion of tumor angiogenetic factors (Li H. et al., 2018). Meanwhile, ginsenoside Rp1 was shown to inhibit murine macrophage radiation-induced DNA damage. After colon cancer cells were exposed to conditioned medium from radiation-potentiated macrophages, it was found that culture supernatant of Rp1-treated macrophages inhibited growth and metastasis of tumor cells and prolonged survival of tumor-bearing mice *in vivo* (Baik et al., 2020). In yet another study, treatment of RAW264.7 cells, a macrophage-derived cell line, with heat-processed ginseng (HPG) containing Rg3, Rk1, and Rg5 that enhanced macrophage cell functions that included cytokines production, MHC class I and II expression, and NF-κB transcriptional activity (Shin et al., 2018).

Another component of ginseng, polysaccharides, are recognized as immunomodulators (Zeng et al., 2019; Qi et al., 2021). It has shown that the addition of red ginseng acidic polysaccharide (RGAP) or IFN-γ alone to murine melanoma B16 cells exerted no cytotoxic effect, while each weakly activated macrophages. It is worth noting that RGAP administered with IFN-γ markedly stimulated macrophages to secrete pro-inflammatory cytokines resembling those secreted by M1 macrophages (e.g., IL-1, IL-6, TNF-α) due to triggering of the NF-κB pathway, leading to dramatic enhancement of MHC-unrestricted macrophage-mediated cytotoxicity (Choi et al., 2008). The non-selective cytotoxicity of most chemotherapeutic drugs, while achieving desired anti-tumor effects, can lead to collateral damage of immune cells, prompting researchers to investigate ginseng effects for alleviating immune cell damage. In one such study, ginseng neutral polysaccharides were shown to reverse 5-fluorouracil-induced splenic weight decreases and inhibition of macrophage phagocytosis to restore macrophage production of NO and TNF-α (Ni et al., 2010). Therefore, ginseng active ingredients appear to have the potential to induce macrophages to transform into pro-inflammatory cells with heightened ability to kill tumor cells, while also potentially alleviating macrophage damage caused by effects of radiotherapy and chemotherapy.

#### Dendritic Cells

Dendritic cells (DCs), which function as professional antigen presenting cells (APCs), play pivotal roles in initiating innate and adaptive immunity, with the latter role associated with DCs presentation of exogenous tumor-associated antigens on MHC I molecules to naive CD8^+^ T cells to initiate anti-tumor immunity. However, the activity of DCs to induce anti-tumor responses is suppressed by TME dampening of DC maturation, differentiation, or cell migration, with numerous TME effector molecules (e.g., IL-6, IL-10, VEGF, TGF-β, CSF-1) involved in suppression of DC activities (Fu and Jiang, 2018; Bandola-Simon and Roche, 2019; Del Prete et al., 2020). Defective DCs that cannot properly perform their sentinel function have been detected in various cancers, such as breast cancer (Gervais et al., 2005), colorectal cancer (Legitimo et al., 2014), and ovarian cancer (Jiang et al., 2018). Increased immunogenic tumor cell death may contribute to maturation and tumor antigen-presentation activity of DCs resulting from release of damage-associated molecular patterns (DAMPs) molecules from tumor cells, such as chaperone protein calreticulin (CRT), high mobility group box-1 protein (HMGB1), and heat shock proteins (HSPs). Keum-joo Son *et al.* (Son et al., 2016) reported that ginsenoside Rg3 was able to act as an immunomodulator to increase DC uptake of tumor cells by inducing immunogenic tumor cell death and enhancing immunogenicity of cancer cells. Furthermore, ginsenoside Rg3 could stimulate tumor cells to produce IFN-γ, an anti-tumor cytokine secreted by T cells, while suppressing tumor cell secretion of TGF-β and IL-6 (Son et al., 2016). In other work, Takei *et al.* (Takei et al., 2004) demonstrated that M1 and M4, end products of steroidal ginseng saponins metabolized within the digestive tract, exerted immunomodulatory effects on DCs by inducing DCs maturation, as reflected by upregulation of DC surface expression of maturation marker molecules CD80, CD83, CD86, and HLA-DR. In turn, mature DCs enhanced the polarization of Th1 cells that increased anti-tumor immunity. In addition, ginseng polysaccharides have been shown to stimulate maturation of murine bone marrow dendritic cells (BMDCs), as revealed by changes in cell morphology, upregulation of membrane phenotypic markers (e.g., CD40, CD80, CD83, CD86, MHC II), and increased pro-inflammatory cytokine production (Meng et al., 2013; Wang et al., 2013).

#### Natural Killer Cells

As for DCs, natural killer (NK) cells in the TME are also dysfunctional (Round and Mazmanian, 2010). Results of studies based on mouse models of lymphoma clearance and metastatic melanoma demonstrated that ginsenoside F1 could enhance NK cell cytotoxicity against diverse types of cancer cells, while also improving NK cell cancer surveillance ability by upregulating NK cell secretion of cytotoxic mediators and NK activation signal molecules (Kwon et al., 2018). Meanwhile, oral administration of 20(S)-protopanaxatriol (M4), an intestinal bacterial metabolite of ginsenosides, led to complete absorption of M4 by the small intestine followed by transfer of most of the substance to the mesenteric lymphatics, where it was esterified to form EM4 that then spread to other organs in the body. Notably, EM4 was shown to stimulate tumor lysis mediated by NK cells in a B16-BL6 melanoma metastasis mouse model (Hasegawa et al., 2002). In another study, pectin polysaccharide fraction (GS-P) purified from ginseng leaves was shown to inhibit proliferation and metastasis of colon cancer cells; these effects were not based on direct cytotoxicity, but were instead based on stimulation of macrophage and NK cell activities (Shin et al., 2017). A similar conclusion was obtained in a study of ginseng berry polysaccharide portion (GBPP), which stimulated macrophages to secrete anti-tumorigenic cytokines (e.g., IL-6, IL-12, TNF-α) while also promoting NK cells to release IFN-γ and granzyme B that inhibited tumor cells activities (Lee et al., 2019a; Lee et al., 2019b). Moreover, another study of polysaccharides purified from ginseng fruits demonstrated that ginseng polysaccharides could significantly enhance NK cell-mediated cytotoxicity in tumor-bearing mice (Wang et al., 2015). Taken together, these results indicate that active components of ginseng exert anti-tumor effects by correcting impaired NK cell killing of tumor cells that, in turn, effectively inhibit tumor metastasis.

### Ginseng Enhancement of the Adaptive Immune Response

#### Adaptive Immune Cells

In addition to ginseng effects for reversing tumor inhibition of innate immune cell activities, ginseng has also been shown to reverse tumor-inhibited adaptive immune cell activities. For example, regulatory T cells (Tregs) (identified based on the presence of foxp3+CD25+CD4^+^ cell surface markers) actively engage in maintenance of immunological self-tolerance, as well as in inhibition of immune responses within the TME (Dong et al., 2020). In fact, Tregs have been found to infiltrate the tumor site, where they suppress the anti-tumor response, with suppression reversed by depletion of Tregs. Using chronic intestinal inflammation as a model system of colorectal cancer (due to the close clinical relationship between the two diseases), ginseng berry polysaccharide extract (GBPE) and GBPP obtained from Asian ginseng berries were shown to exert anti-inflammatory effects *in vitro* that were linked to inhibition of secretion of IL-8, a proinflammatory factor closely tied to intestinal inflammation. Moreover, by inhibiting T cell differentiation into Th1 cells (which promote intestinal inflammation) and Treg cells (which weaken the body’s anti-tumor immunity), intestinal inflammation may be reduced and anti-tumor effects of chemotherapy drugs synergistically enhanced so as to effectively inhibit proliferation of colorectal cancer cells (Wang et al., 2020).

**FIGURE 2:**
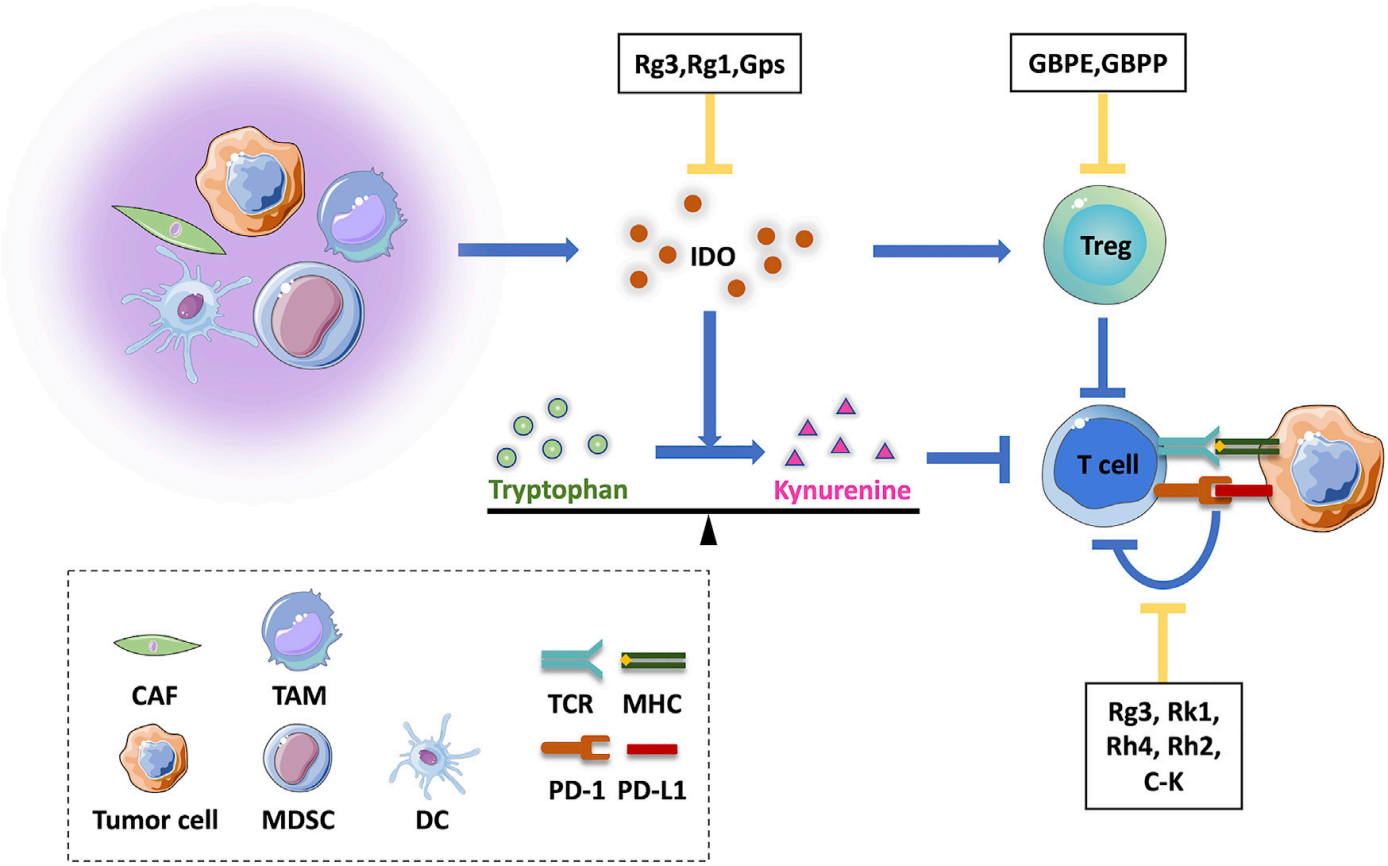
Effects of active ingredients of ginseng on acquired immune responses in the tumor microenvironment. A variety of cells in the tumor microenvironment may inhibit T cell functions, either through direct modulation of the IDO molecular switch or indirect effects that maintain immunosuppressive Treg cells within the tumor microenvironment. Tumor cells inhibit T cell functions through the PD-1/PD-L1 axis, resulting in immune escape. Active ingredients of ginseng act to inhibit these processes. CAF, cancer-associated fibroblast; TAM, tumor-associated macrophage; MDSC, myeloid-derived suppressor cell; DC, dendritic cell; GBPE, ginseng berry polysaccharide extract; GBPP, ginseng berry polysaccharide portion; IDO, indoleamine 2,3-dioxygenase.

Indoleamine-2,3-dioxygenase (IDO) is an intracellular heme-containing enzyme within the kynurenine (Kyn) pathway that catabolizes tryptophan (Trp). Due to its role in tumor immune escape mechanisms, IDO expression has been found in tumor cells, DC cells, endothelial cells and even stromal cells in the TME, where it inhibits CTL infiltration and cell functions while inducing Treg recruitment (Uyttenhove et al., 2003; Löb et al., 2009; Sharma et al., 2010; Munn and Mellor, 2016). Using the ratio of Kyn to Trp as a marker of IDO enzymatic activity, results of a study based on a mouse model of inflammation indicated that ginseng total saponins could decrease the plasma Kyn/Trp ratio (Kang et al., 2011). Since then, researchers have also found that ginsenoside Rg3 treatment similarly reduced IDO activity in the periphery and brain (Kang et al., 2017). In an *in vivo* liver fibrosis mouse model and in *in vitro* experiments, ginsenoside Rg1 was also observed to inhibit IDO1 protein expression and enhance DCs activities and T cell infiltration, thereby enhancing the immune response (Mo et al., 2021). Recent studies have shown that ginseng polysaccharides enhanced the anti-tumor response triggered by anti-PD-1 mAb by increasing CD8^+^ T cell function and decreasing Treg inhibition. These effects may have been due to effects of ginseng polysaccharides on tryptophan metabolism that acted to reshape the gut microbiota that resulted in significantly increased production of L-tryptophan and decreased production of L-kynurenine and IDO expression in tumor cells (Huang et al., 2021). In conclusion, ginsenosides and ginseng polysaccharides appear to enhance the adaptive immune response against tumor cells, warranting further study (Figure 2).

#### Enhancement of Immunogenicity of Tumor Cells

Immunogenicity of tumor cells is key to inducing an anti-tumor immune response. Generally, cancer cells could evade anti-tumor immunity by adopting active immunogenicity reduction strategies, including reduced expression of tumor antigens, diminished MHC-I expression for reduced antigenic recognition by T cells, and aberrant expression of immune checkpoint proteins, such as programmed death ligand-1 (PD-L1), which inhibits existing host anti-tumor immunity (Kim and Seo, 2018; Looi et al., 2019; Tang et al., 2014). Furthermore, PD-L1 expression can be up-regulated in tumor cells that are resistant to chemotherapeutic agents (Shen et al., 2019; Wu et al., 2021), including tumor-targeting drugs such as EGFR-TKI (Peng et al., 2019), thus promoting immune escape of tumor cells. Although inhibitors targeting immune checkpoints have been developed and have become a focal point of clinical cancer therapy, current low treatment response rates, high incidence of side-effects, and acquired resistance are still unavoidable challenges. Nevertheless, effective reduction of PD-L1 expression in tumor cells appears to anti-tumor immunity and reduce tumor drug resistance, as shown in a study whereby PD-L1 protein was overexpressed in cisplatin-resistant human non-small cell lung cancer (NSCLC) cell line A549/DDP. In these experiments, ginsenoside Rg3 was shown to significantly block PD-L1 overexpression in A549/DDP cells, an effect that was associated with inhibition of activation of Akt and NK-κB pathways. Additional *in vitro* experiments confirmed that when A549/DDP cells were co-cultured with CD8^+^ T cells, Rg3 could target chemotherapy-induced PD-L1 and enhance the immunocytotoxicity of CD8^+^ T cells (Jiang et al., 2017). Moreover, when loaded into carbon nanotubes (CNT) (Luo et al., 2021), Rg3 inhibited expression of PD-L1 by triple-negative breast cancer cells. Remarkably, CNTs-loaded with Rg3 could attenuate PD-1 expression in activated T cells and reduce PD-1/PD-L1 axis activity *in vitro*. Meanwhile, other ginsenosides such as Rk1 (Hu et al., 2020), Rh4 (Deng et al., 2020), and Rh2 (Chen Y. et al., 2020) have also been reported to downregulate PD-L1 expression in tumor cells (Figure 2). In addition, ginsenosides have been shown to exert a competitive inhibitory effect on the PD-1/PD-L1 interaction. Specifically, eight ginsenosides (Rd, F2, Rg3, C-K, Rh2, PPD, Rg1, Rh1) were confirmed to block the PD-1/PD-L1 binding interaction, whereby a blocking ability of 35% was observed at the maximum ginsenoside concentration (1 µM), with Rg3 and C-K exerting the most significant effects. Further analyses based on molecular docking and pharmacophore analysis suggested that Rg3 and C-K may achieve an immune checkpoint blockade through multiple hydrophobic and hydrogen bonds with PD-1/PD-L1 molecules (Yim et al., 2020).

## Ginseng Could Inhibit Stemness of Cancer Stem Cells

In recent decades, rare stem-like cells known as cancer stem cells (CSCs) have been detected in tumors. CSCs possess characteristics of self-renewal, strong xenograft tumorigenesis properties, and resistance to conventional therapy (radiation and chemotherapy) and resemble bone marrow hematopoietic stem cells. In general, CSCs are dormant except during long-term tumor growth, where they can become activated to engage in self-renewal and differentiation to generate heterogeneous tumor cells with epithelial-mesenchymal transition (EMT) characteristics (López de Andrés et al., 2020). CSCs have been identified using various identification methods, including *in vivo* limiting-dilution tumorigenicity assays in immunodeficient mice, *in vitro* tumorsphere formation assays, and assays to detect particular surface markers (Walcher et al., 2020). In multiple types of solid tumors, such as breast cancer (Bai et al., 2018), lung cancer (Heng et al., 2019), brain cancer (Singh et al., 2003), and colon cancer (Gupta et al., 2019), CSCs have been identified and found to play crucial roles in tumor proliferation, metastasis, relapse, and chemotherapy/radiation resistance resulting in failures of anti-cancer therapies. Maintenance of CSCs is dependent on TME characteristics such that the acidic and hypoxic environment of the TME may support the establishment and maintenance of CSCs stemness properties, while initiating and regulating stem cell-like programs through various developmental signaling pathways. Such pathways are crucial for maintenance of stem and progenitor cell homeostasis and functions and include Norch, WNT, Hedgehog, and Hippo pathways (Meurette and Mehlen, 2018; Clara et al., 2020). Recent studies have found that endothelial cells/pericytes within some tumor vessels may be generated via differentiation from CSCs (Krishna Priya et al., 2016), thus highlighting CSCs as a novel promising target for use in regulating the TME.

Ginseng has been reported to decrease the size of the CSCs population (Table 3). A Korean research team fermented red ginseng with *Lactobacillus rhamnosus* KCTC 5033 (f-RGE) to increase Rg3 content level and found that f-RGE had the potential to inhibit differentiation of breast cancer stem cell-like cells in the presence of carcinogens (Oh et al., 2015). It was further demonstrated that *in vitro*, Rg3 treatment could reduce the size of the population of CD24+/CD44+/EpCAM+ colon cancer stem cells and inhibit their clone-forming ability, with similar results obtained from an *in vivo* orthotopic xenograft model study (Tang et al., 2018) and a breast cancer study (Oh et al., 2019). In addition, ginseng had the potential to modulate the CSC phenotype. For example, BST204, a fermented ginseng extract containing high quantities of Rh2 and Rg3, strongly suppressed cancer stemness of embryonic carcinoma cells by downregulating stemness markers and transcription factors at both transcriptional and protein expression levels (Park et al., 2020). Meanwhile, ginsenoside Rg3 was also shown to decrease tumor CSC sphere-forming capacity that led to deregulated expression of stemness-related markers and attenuated CSC tumorigenic activities in several types of cancer (Wang et al., 2018; Phi et al., 2019a; Oh et al., 2019; Tan et al., 2020). Moreover, it has been reported that ginsenoside Rd may downregulate levels of genes related to stemness and EMT by binding to epidermal growth factor receptor (EGFR) (Phi et al., 2019b). Additionally, ginsenosides Rk1 and Rg5 have also been reported to suppress expression levels of lung CSC surface markers CD44 and CD133 and transcriptional regulators Nanog, Oct4, and Sox2 (Kim et al., 2021). Furthermore, the inhibitory effect of ginsenosides on stemness of CSCs has been shown to increase cell sensitivity to chemotherapeutic treatments while also reducing resistance to chemotherapy (Tang et al., 2018; Wang et al., 2018; Tan et al., 2020). At present, mechanisms whereby ginsenosides regulate CSCs are still unclear, while results regarding effects of ginsenosides on CSCs in the TME and effects of other ginseng active ingredients on CSCs await future verification. However, from the body of accumulated data, it is apparent that ginseng holds great promise as a drug for targeting CSCs.

**TABLE 3:** Effects of ginseng on cancer stem cells.

Components	Cell/animal model	Effects	Main mechanisms	Ref.
Fermented red ginseng with L. rhamnosus KCTC 5033	*In vitro*, mimicking breast cancer stem cells MCF-7	Could reduce the viability of reprogrammed MCF-7 cancer stem-like cells	Not clear	Oh et al. (2015)
Ginsenoside Rg3	*In vitro*, colorectal cancer cells(CRC) LoVo. *In vivo*, CRC cells LoVo orthotopic xenografts	Could repress the growth and migration CRC cells and strengthen the cytotoxicity of 5-Fu and oxaliplatin	Partly depend on decreasing proportion of stem cells expressing CD24+/CD44+/EpCAM+ and attenuate the stemness of CRC cells	Tang et al. (2018)
Ginsenoside Rg3	*In vitro*, stem-like NSCLC cells H1975, Osimertinib-resistant H1975 cells(H1975-OR)	Could decrease spheroid formation ability, expression of stemness-related markers, ALDH activity, and attenuate the Osimertinib resistance of NSCLC cells	Activating Hippo signaling	Tan et al. (2020)
Ginsenoside Rg3	*In vitro* and *in vivo*, stem-like NSCLC cells H1299 and A549	Could inhibit spheroid formation ability, expression of stemness-related markers, sensitize hypoxic NSCLC cells to cisplatin	Inhibiting NF-kB signaling pathway	Wang et al. (2018)
20(R)-Ginsenoside Rg3	*In vitro*, CRC cells HT29 and SW620	Could downregulate the levels of stemness genes and EMT markers	Inhibiting EGFR/SNAIL signaling	Phi et al. (2019a)
Standardized Korean Red Ginseng extract (RGE), ginsenoside Rg3	*In vitro*, stem-like breast cancer cells MCF-7 and MDA-MB-231	Could decrease the viability, number and the size of mammospheres, proportion of CD44^high^/CD24^low^ CSCs and ALDH positive cells, and reduce the expression of self-renewal signaling molecules	Partially dependent on the PI3K/Akt pathway	Oh et al. (2019)
Fermented ginseng extract BST204	*In vitro*, embryonic carcinoma cells NCCIT	Could downregulate the levels of stemness and stem-related transcription factors genes, and inhibit CSCs tumorigenesis	Target CD133	Park et al. (2020)
Ginsenoside Rd	*In vitro*, CSC-like CRC cells HT29 and SW620	Could suppress the growth of CSCs, and downregulate expression levels of CSC markers.	Inhibiting EGFR/Akt signaling	Phi et al. (2019b)
Ginsenoside Rg5 and Rk1	*In vitro*, human NSCLC cells A549	Could inhibit tumorsphere formation ability, suppressed the stem cell-like properties	Suppressing Smad2/3, NF-kB, ERK, p38 MAPK, and JNK pathways	Kim et al. (2021)

## Conclusion

The TME, a complex ecological system composed of a variety of cells and stroma, is a hotbed of tumor development. Tumor cell interactions with the TME lead to greater tumor cell aggressiveness and resistance to conventional drugs and radiation. Therefore, anti-tumor therapies should both eliminate tumor cells and interfere with communication between tumor cells and the TME.

Due to the fact that targeting of the TME is a promising anti-cancer strategy, agents that target TME inputs hold great promise as future anti-cancer drug treatments. Such agents in current use include monoclonal antibodies against three key targets: immune checkpoints PD-1 and CTLA-4, which enhance tumor-killing T cell immune functions; pro-angiogenic factor VEGF; and integrin adhesion molecules that mediate interactions between tumor cells and the TME matrix. However, anti-cancer drugs inevitably have side effects and development of new anti-cancer compounds and antibodies is extremely expensive, although screening of databases containing information for a huge array of natural compounds is a relatively inexpensive and effective approach for identifying potential anti-cancer drugs.

Ginseng is a versatile natural herbal medicine that has been shown to exert good anti-inflammatory, antioxidant, and anti-aging therapeutic effects. In fact, ginsenosides, which are mainly found in ginseng root, are considered to be the most important biologically active components of ginseng preparations. So far, more than 180 types of ginsenosides have been isolated from ginseng (Xu et al., 2021). In addition, another active ingredient of ginseng, polysaccharides (the structure and extraction method of polysaccharides are listed in Table 4), also possess beneficial activities for modulating immune regulatory functions. Inhibitory effects of some ginsenoside monomers and ginseng polysaccharides on cancer cell activities have been studied, with regulatory effects of ginseng on TME under increasing scrutiny. In this review, ginseng effects on tumor angiogenesis, the tumor immunosuppressive microenvironment, and tumor stem cells are summarized.

**TABLE 4:** Structure and extraction method of polysaccharide from ginseng.

Polysaccharide	Extraction and separation method	Composition sugar ratio (Mass percentage or molar ratio)	Molecular weight	Ref.
Red ginseng acidic polysaccharide (RGAP)	Distilled water percolation, precipitation by 85% ethanol, purification by dialysis (15 kDa)	GlcA: Glc: GalA = 51.8: 26.1: 5.1	>15 kDa	Choi et al. (2008)
Ginseng neutral polysaccharide fraction	Hot water exaction, precipitation by 95% ethanol, purification by Sevage and DEAE -Cellulose	Glc: Gal: Ara = 95.3%: 3.3%: 1.3%	_	Ni et al. (2010)	
Neutral ginseng polysaccharides (NGP)	NGP bought from Pude Pharmaceutical Company	Main ingredients is α-(1→6)-D-Glucan	504 Da	Meng et al. (2013)	
Ginseng polysaccharides	GPS injection was bought from Shanxi Pude pharmaceutical Co., Ltd., Shanxi, China	_	_	Ma et al. (2014)	
Acidic ginseng polysaccharides (AGP)	AGP (>99% purity, 3 mg/ml) was bought from Pude pharmaceutical company, Shanxi, China	Sugar of composition are GalA, Glc, Ara, Xyl and Rha	66 kDa	Wang et al. (2013)	
Ginseng berry polysaccharide portion (GBPP)	Hot water exaction, precipitation by 95% ethanol, purification by dialysis (20 kDa)	Rha: Fuc: Ara: Xyl: Man: GalA: Glc = 8.4: 19.5: 2.2: 1.5: 39.8: 5.4	>20 kDa	Lee et al. (2019a)	
GBPP-I	Hot water exaction, precipitation by 90% ethanol, purification by dialysis (20 kDa) and G-75 gel permeation column	Glc: GalA: Gal: Ara: Rha = 5.4: 10.4: 46.9: 27.5: 6.7	76 kDa	Lee et al. (2019b)	
Ginseng leaves polysaccharide fraction	Hot water exaction, purification by Diaion HP-20 column and Diaion PA312 column, precipitation by 95% ethanol and dialysis (1000Da)	Rha: Fuc: Ara: Gal: GalA: GlcA: Man: Xyl = 10.2: 3.1: 14.4: 11.8: 37.3: 2.5: 0.7: 0.9	10.2 kDa	Shin et al. (2017)	
Ginseng fruits polysaccharide	Hot water exaction, precipitation by 95% ethanol, purification by Sevage, DEAE-cellulose-52 and Sepharose CL-6B column	Gal: Glc: Rha: Ara = 6.1: 2.0: 1.1: 3.2	140 KDa	Wang et al. (2015)	

The gut microbiota maintains a symbiotic relationship with intestinal mucosa, the largest immune organ of the human body. The importance of this symbiotic interaction to host well-being is based on its ability to shape the host immune system by regulating local and systemic immune responses. For example, gut microbiota and metabolites may activate DCs in the local intestine, thereby activating the transformation of primitive T cells into effector T cells in mesenteric lymph nodes, with special importance for development of Treg and Th17 cells (Round and Mazmanian, 2010; Cheng et al., 2019). Alternatively, metabolites of the gut microbiota could also enter the blood circulation, thus affecting the immune function of the entire body (Rooks and Garrett, 2016; Ohno, 2020). A large body of experimental evidence suggests that gut microbes influence tumorigenesis and functions of immune cells in the TME by regulating production of cytokines (De Almeida et al., 2019; Untersmayr et al., 2019; Li Q. et al., 2020; Chen et al., 2021). In addition, gut microbes are closely associated with the efficacy and toxicity of anti-tumor immunotherapies, such as adoptive cell transfer therapy (Viaud et al., 2013; Nelson et al., 2015; Uribe-Herranz et al., 2018) and immune checkpoint inhibitors (Vétizou et al., 2015; Gopalakrishnan et al., 2018; Routy et al., 2018; Elkrief et al., 2019). Therefore, the gut microbiota not only serves as a new observational index of tumor immunotherapy, but also holds promise as an anti-tumor therapeutic target. In accordance with these concepts, previous studies indicated that the efficacy of oral ginseng was related to gut microbiome-mediated metabolic transformation involving two types of ginsenoside biotransformation: conversion of protopanaxadiol-type ginsenosides to form compound K and ginsenoside Rh2; conversion of protopanaxatriol-type ginsenosides to form ginsenosides Rh1 and protopanaxatriol (Kim, 2018). Interestingly, ginsenosides and ginseng polysaccharides have also been used to regulate the structure of the gut microbiome for treating a variety of diseases, such as obesity (Song et al., 2014; Lee et al., 2021), diabetes (Li J. et al., 2018; Xu et al., 2020), colitis (Wang et al., 2016; Chen L. et al., 2020), and antibiotic-related diarrhea (Qu et al., 2021). With regard to cancer, a recent study showed that oral ginseng polysaccharides combined with anti-PD-1-mAb could improve therapeutic sensitivity of anti-PD-1-mAb in patients with NSCLC. This effect may have been related to ginseng polysaccharides-induced remodeling of gut microbiota structure in chemotherapeutic non-responders that led to increased abundance of metabolites, such as short-chain fatty acids (SCFAs), while also down-regulating IDO activity (Huang et al., 2021). Thus, the immunosuppressive TME associated with NSCLC was altered, leading to heightened immunotherapeutic sensitivity induced by ginseng polysaccharides administration. Consequently, we hypothesized that the regulatory effects of active ingredients of ginseng on anti-tumor immunity and on the tumor immunosuppressive microenvironment may be partly related to the gut microbiome, although we have found only a few published reports describing such an association. Nevertheless, interrelationships between the regulation of gut microbiome by active ginseng ingredients, the tumor immune microenvironment, and tumor immunotherapeutic effects are unknown and require additional evidence, warranting further research.

In order to improve organ/tumor-site targeting, increase solubility of ginseng active ingredients, and reduce drug toxicity toward non-cancerous cells, researchers have combined nanoscale drug delivery systems with ginseng-derived drugs to improve biological activities of ginseng active ingredients for enhanced therapeutic effect. For example, a folic acid-modified targeting-drug delivery system based on bovine serum albumin nanoparticles achieved targeted accumulation of drugs at the cancer focus that significantly increased anti-cancer effectiveness of Rg5 in breast cancer (Dong et al., 2019). Meanwhile, use of multiple nanoparticle-loaded Rg3 was shown to achieve good organ targeting, possess sustained release properties, and exert superior anti-cancer activities, while also facilitating transport of drugs across the blood-brain barrier (Qiu et al., 2019; Ren et al., 2020; Su et al., 2020). These results support potential benefits of ginseng ingredients for use in numerous clinical applications.

**FIGURE 3:**
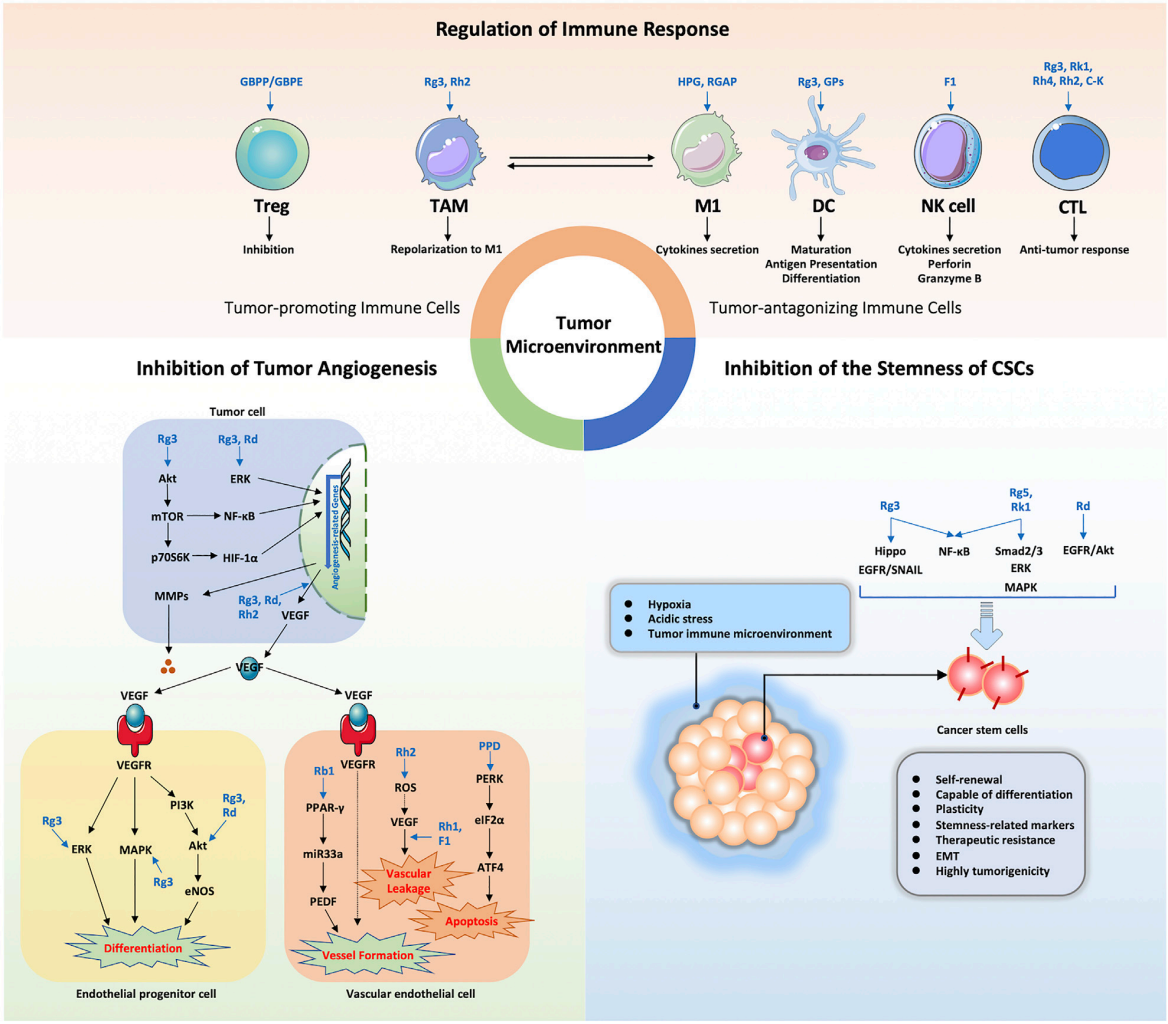
Summary of the functional effects and mechanisms underlying TME regulation by ginseng active ingredients via multiple targets. HPG, heat-processed ginseng; RGAP, red ginseng acidic polysaccharide; GPs, ginseng polysaccharides; NK cell, natural killer cell; CTL, cytotoxic T lymphocyte.

Although targets of ginseng anti-cancer effects are unknown, it is undeniable that active ingredients of ginseng influence the interaction between the tumor and the TME through several mechanisms: by inhibiting tumor angiogenesis, regulating the immunosuppressive TME, and by inhibiting stemness of cancer stem cells (Figure 3). Therefore, use of a combination of ginsenosides and/or polysaccharides as cancer adjuvant therapies to target the TME may be a useful anti-tumor therapeutic strategy that may also reduce side effects of chemotherapy or immunotherapy. In addition, the application of red ginseng and white ginseng roots for adjuvant treatment of tumor patients is practical, but the dose must be further standardized and validated using clinical data and the ginseng target network must be further elucidated. In conclusion, this review provides new insights into possible applications of active ingredients of ginseng for achieving TME remodeling.
